# Isolation and characterization mesenchymal stem cells from red panda (*Ailurus fulgens styani*) endometrium

**DOI:** 10.1093/conphys/coac004

**Published:** 2022-02-21

**Authors:** Dong-Hui Wang, Xue-Mei Wu, Jia-Song Chen, Zhi-Gang Cai, Jun-Hui An, Ming-Yue Zhang, Yuan Li, Fei-Ping Li, Rong Hou, Yu-Liang Liu

**Affiliations:** 1 Chengdu Research Base of Giant Panda Breeding, 1375 Panda Road, Northern Suburb, Chengdu, 610081, Sichuan Province, China; 2 Sichuan Key Laboratory of Conservation Biology for Endangered Wildlife, 1375 Panda Road, Northern Suburb, Chengdu, 610081, Sichuan Province, China; 3 Sichuan Academy of Giant Panda, 1375 Panda Road, Northern Suburb, Chengdu, 610081, Sichuan Province, China

**Keywords:** red panda, mesenchymal stem cell, endometrium, cell differentiation, Cell culture

## Abstract

Endometrial mesenchymal stem cells (eMSCs) are undifferentiated endometrial cells with self-renewal, multidirectional differentiation and high proliferation potential. Nowadays, eMSCs have been found in a few species, but it has never been reported in endangered wild animals, especially the red panda. In this study, we successfully isolated and characterized the eMSCs derived from red panda. Red panda eMSCs were fibroblast-like, had a strong proliferative potential and a stable chromosome number. Pluripotency genes including *Klf4*, *Sox2* and *Thy1* were highly expressed in eMSCs. Besides, cultured eMSCs were positive for MSC markers CD44, CD49f and CD105 and negative for endothelial cell marker CD31 and haematopoietic cell marker CD34. Moreover, no reference RNA-seq was used to analyse the eMSCs transcriptional expression profile and key pathways. Compared with skin fibroblast cell group, 9104 differentially expressed genes (DEGs) were identified, among which are 5034 genes upregulated, 4070 genes downregulated and the top 20 enrichment pathways of DEGs in Gene Ontology (GO) and the Kyoto Encyclopedia of Genes Genomes (KEGG) mainly associated with G-protein coupled receptor signalling pathway, carbohydrate derivative binding, nucleoside binding, ribosome biogenesis, cell cycle, DNA replication, Ras signalling pathway and purine metabolism. Among the DEGs, some representative genes about promoting MSCs differentiation and proliferation were upregulated and promoting fibroblasts proliferation were downregulated in eMSCs group. Red panda eMSCs also had multiple differentiation ability and could differentiate into adipocytes, chondrocytes and hepatocytes. In conclusion, we, for the first time, isolated and characterized the red panda eMSCs with ability of multiplication and multilineage differentiation *in vitro*. The new multipotential stem cell could be beneficial not only for the germ plasm resources conservation of red panda, but also for basic or pre-clinical studies in the future.

## Teaser

In this work, we, for the first time, isolated and characterized the endometrial mesenchymal stem cells from red panda. It could be beneficial not only for the germ plasm resources conservation, but also for basic or pre-clinical studies (e.g. the study of interspecies somatic cell nuclear transfer and embryonic diapause) in the future.

## Introduction

Red pandas (*Ailurus fulgens*), world-famous wild animals, are mainly distributed in the west of China, the Himalaya mountain ranges of Nepal, India, Bhutan and Myanmar (Li *et al.,* 2005). The red pandas are recently classified as two separate species *Ailurus fulgens fulgens* and *Ailurus fulgens styani*. In China, both subspecies are found and were separated by Yalu Zangbu River (Hu *et al.,* 2020). Due to the destructed habitats and declining population, the red panda was listed as endangered by the International Union for Conservation of Nature (IUCN) Red List of Threatened Species in 2015 (Glatston *et al.,* 2015). Nowadays, some measures such as *ex situ* and *in situ* conservations were taken to protect red pandas. In addition, the efficient preservation and utilization of stem cell resources is also an important way to protect endangered wildlife (Stanton *et al.,* 2019). Assisted reproduction combined with embryonic stem cell technology also played a valuable role in the effort to protect endangered species (Hildebrandt *et al.,* 2018; Saragusty *et al.,* 2020).

Mesenchymal stem cells (MSCs), firstly identified in the bone marrow, are a population of pluripotent cells with high proliferative rate, low immunogenicity, self-renewal and multi-directional differentiation potential throughout the entire stage of life (Friedenstein *et al.,* 1966; Li *et al.,* 2015; Mason *et al.,* 2014). In humans, MSCs have been identified in many tissues including umbilical cord blood and adipose tissues (Lv *et al.,* 2014). As for wild animals, earlier studies by our group have identified the giant panda bone marrow MSCs (Liu *et al.,* 2013) and umbilical cord MSCs (Liu *et al.,* 2021), as well as the red panda bone marrow MSCs for the first time (An *et al.,* 2020).

In addition to the above-mentioned tissues, endometrium contains a small population of cells with typical MSC properties (Gargett *et al.,* 2009). Endometrial MSCs (eMSCs) expressed cell surface markers, including CD29, CD44, CD73, CD90 and CD105 (Du *et al.,* 2016; Gargett & Masuda, 2010; Murphy *et al.,* 2008), had the ability to differentiate into adipocytes, osteoblasts, chondrocytes and smooth muscle cells *in vitro* (Rink *et al.,* 2017). Moreover, eMSCs could generate endometrial stroma in xenograft assays (Masuda *et al.,* 2010). eMSCs have been harvested and characterized from humans (Cheng *et al.,* 2017; Queckborner *et al.,* 2020), pigs (Miernik & Karasinski, 2012), heifers (Calle *et al.,* 2019; de Moraes *et al.,* 2016), horses (Cabezas *et al.,* 2018), dogs (De Cesaris *et al.,* 2017; Sahoo *et al.,* 2017) and sheep (Ghobadi *et al.,* 2018). This type of cells showed similar properties to bone marrow MSCs and may provide an available source for cell-based therapies due to their strong regenerative capacity (Gargett *et al.,* 2009). The therapeutic potential of eMSCs has already been demonstrated in relation to premature ovarian failure (Lai *et al.,* 2015), Parkinson’s disease (Wolff *et al.,* 2011), pelvic organ prolapse (Emmerson & Gargett, 2016) and angiogenesis (Pence *et al.,* 2015).

Recently, *in vitro* gametogenesis was proposed as the ultimate solution for infertility caused by loss or compromised function of gametes (Makar & Sasaki, 2020). Reconstitution of primordial germ cell (PGC) specification from pluripotent cells is an essential first step for *in vitro* gametogenesis. Previous study reported that PGC-like cells were successfully derived from canine adipose mesenchymal stem cells (Wei *et al.,* 2016). Human amniotic membrane MSCs could be induced to express PGC gene markers and have enough potential to PGC specification (Alifi & Asgari, 2020). During uterine organogenesis, cell communications were closer and polypotential germ cells differentiated and grew into myometrium and endometrial layers (Makiyan, 2017). Moreover, endometrium is the site for embryo implantation and accompanies all stages of post-implantation embryo development and has a direct intercellular communication with the embryo (Massimiani *et al.,* 2019). Early studies in bovine have found that eMSCs ensured the maternal immunomodulation required for embryo survival (Calle *et al.,* 2019). Therefore, the eMSCs would have the potential to PGC specification, *in vitro* gametogenesis and embryo implantation regulation.

For endangered wild animals, cell germ plasm resources are extremely precious, which is one of the key factors to protect the genetic diversity of species. In addition, the research on the reproductive mechanism of wild animals is not well explained and the potential regulation should be further studied.

Therefore, the aim of this study was to isolate and characterize MSCs from red panda endometrium. The new type of cells will be beneficial for germ plasm resources conservation of red panda, as well as for basic or pre-clinical studies in the future.

## Material and methods

### Isolation of eMSCs from red pandas

The samples used in this study were obtained postmortem from six red pandas (Supplementary Table S1), which were raised in the Chengdu Research Base of Giant Panda Breeding. All eMSCs isolation performance of the six red pandas were performed as follows. Briefly, the harvested uterus samples were washed five times in phosphate-buffered saline (PBS, Gibco) with 5% antibiotic–antimycotic solution (Gibco). Uteruses were cut into 1 × 1 × 1 mm pieces without fatty tissues under sterile condition, and then washed twice in PBS. The uteruses were mechanically minced and dissociated into single cells with collagenase type IV (1 mg/ml; Gibco) for 15 min at 37°C, then centrifuged at 600 g for 3 min. Discarding the supernatant, the tissue was further digested with 0.25% trypsin (Gibco) for 15 min at 37°C. Cell suspensions were filtered through a 40-μm sieve (BD Falcon). The filtrates were centrifuged at 600 g for 5 min at room temperature. The cell pellets were resuspended and cultured in culture dishes (diameter, 10 cm; Corning) containing low-glucose Dulbecco’s modified Eagle’s medium (LG-DMEM, Gibco) supplemented with 10% foetal bovine serum (Gibco), 10 ng/ml basic fibroblast growth factor (Peprotech) and 1× antibiotic–antimycotic. The cells were incubated at 37°C with 5% CO_2_, and the medium was changed every 2 days. At ~10 days, the primary eMSCs were passaged with 1× TrypLE Express (Gibco) when the cells reached ~80% confluence.

### Cellular proliferation assay

Red panda eMSCs at passages 4–7 were seeded in 24-well plates at a density of 1 × 10^4^ cells per well to establish the growth curves. The numbers of cells per three wells were counted every day for eight successive days. Cells were counted by automatic cell counter (Countstar) after acridine orange (AO)/propidium Iodide (PI) staining (Countstar).

### Cell surface antigen analysis

Red panda eMSCs at passage 4 were digested with TrypLE Express (Gibco), then washed twice and incubated in buffer with the relevant antibody or with the corresponding isotype control IgG for 40 min. Then cells were washed three times and analysed by using flow cytometry (NovoCyte, ACEA). Compensation and data analysis were performed using FlowJo software (Tree Star, Inc., Ashland, OR, USA). Corresponding antibodies used for flow cytometry analysis were listed in Table 1.

**Table 1:** The antibodies used for flow cytometry

Antibody	Cat. no.	Corresponding isotype antibody	Cat. no.
CD31-PE-Cyanine7	25-0311-81, eBioscience	Rat IgG2a kappa-PE-Cyanine7	25-4321-81, eBioscience
CD34-PE	RM3604, eBioscience	Rat IgG2a kappa-PE	12-4321-80, eBioscience
CD44-PE	12-0441-81, eBioscience	Rat IgG2b kappa-PE	12-4031-82, eBioscience
CD49f-PE	12-0495-81, eBioscience	Rat IgG2a kappa-PE	12-4321-80, eBioscience
CD105-PE	12-1057-42, eBioscience	Mouse IgG1 kappa-PE	12-4714-81, eBioscience

### Karyotype analysis

For karyotype analysis, red panda eMSCs at passage 8 were exposed to 10 ug/ml colcemid (Beyotime) for 4 h, then digested and resuspended in 0.075 M KCl (Sigma) at 37°C for 40 min. After that, eMSCs were fixed in acetic acid and methanol (1:3) (Sigma). The numbers of chromosomes were counted by an inverted fluorescence microscope (BX53, Olympus) with an oil immersion objective. Chromosome images were analysed by ImageJ software (National Institutes of Health, Bethesda, MD, USA).

### RT-PCR

Red panda eMSCs at passage 4 were used for reverse transcription-polymerase chain reaction (RT-PCR) analysis. Total RNA of cultured cells was extracted with the RNAprep Pure Cell Kit (TIANGEN) in accordance with the manufacturer’s instruction. Then, the samples were treated with DNase to remove possible contamination by genomic DNA and reverse transcribed into cDNA using PrimeScript RT reagent Kit (Takara). The specific primer sequences were shown in Table 2, and *β-actin* was used as reference gene.

**Table 2:** Primers used in RT-PCR

Gene	Primer nucleotide sequence (5′ to 3′)	Product size (bp)	Annealing
*Klf4*	F:GTGTCGGGGTAGTCCTGTTG	153	60°C
	R:CCAAGATCAAGCAGGAGGCA		
*Thy1*	F:GCCACGAGAACTCTACCACC	130	60°C
	R:CTGGTGAAGTTGGTTCGGGA		
*Sox2*	F:AACCAGCGCATGGACAGCTA	226	58°C
	R:GCGAGTAGGACATGCTGTAGG		
*CD44*	F:ACAATGGCAATGGAGCGGTA	151	60°C
	R:TTCTGCAGGTTCCGTGTCTC		
*CK18*	F:GCAGATTGAGGAGAGCACCACAG	337	60°C
	R:TTCCAGCAGGCGACGGTAGG		
*ALB*	F:TACGGCGAGCTGGCTGACTG	363	60°C
	R:CTGGAGGCTGGCACACTTGAAC		
*DKK1*	F:CACAGCACCGTGGATGGGTATTC	310	60°C
	R:TCTGACAGGTGTGGAGTCTGGAAG		
*β-actin*	F:ACGATATCGCTGCGCTTGTG	220	60°C
	R:ACAATACCGTGCTCGATGGG		

### RNA-seq and differentially expressed genes enrichment analysis

Red panda eMSCs isolated from three different individuals and corresponding red panda skin fibroblast cells (skin FCs) at passage 4 were seeded in culture dishes (diameter, 10 cm) and treated with normal growth medium. When the cells reached ~80% confluence, the cells were collected and treated with Trizol (Thermo Fisher) as manufacturer’s protocol to extract total RNA and then for RNA-seq (Novogene). The RNA-seq data were assembled and analysed as no reference genome sequences. Differential expression analysis was performed with DESeq2. For functional enrichment analysis, DEGs were mapped to terms in the GO database, and then searched for significantly enriched GO terms (*P* < 0.05). DEGs were mapped to the KEGG database, and searched for significantly enriched KEGG pathways (*P* < 0.05).

### Western blotting

Red panda skin FCs and eMSCs at passage 4 were washed twice with PBS and resuspended in EBC 250 lysis buffer (Beyotime). All other western blotting procedures were conducted as previously reported (An *et al.,* 2020). Briefly, protein concentration was determined by using the protein assay reagent (Bio-Rad). Then, proteins were separated on 4–5% precast gels (Bio-Rad) and electrotransferred to nitrocellulose membranes (Bio-Rad). The membranes were blocked in 5% skim milk diluted in Tris-buffered saline containing 0.1% Tween 20 for 1 h. After blocking, the membranes were incubated overnight at 4°C with rabbit anti-SOX2 (dilution 1:1000) (ab97959, Abcam), rabbit anti-GAPDH (dilution 1:1000) (ab9485, Abcam) and rabbit anti-ALB (dilution 1:1000) (ab207327, Abcam) as primary antibodies. After washing, membranes were incubated with an anti-rabbit HRP-conjugated secondary antibody (dilution 1:5000) (111-035-003, Jackson ImmunoResearch) for subsequent detection by ECL (Millipore). Band intensities were calculated using densitometry in Quantity One software (Bio-Rad).

### Multilineage differentiation

For adipocytic differentiation, eMSCs at passage 4 were seeded in 6-well plates and treated with adipogenic medium (Cyagen) as per the manufacturer’s protocol. The medium was changed three times per week. After 8 days, adipogenesis was evaluated by Oil red O staining (Sigma). Staining was assessed by bright-field inverted microscopy (IX73, Olympus). Red panda eMSCs cultured in normal growth medium served as control.

For chondrogenic differentiation, eMSCs at passage 4 at a density of 4 × 10^5^ cells were cultured in chondrogenic differentiation medium (Cyagen) as per the manufacturer’s protocol. The medium was changed three times per week. After 21 days, chondrogenesis was detected by the staining of toluidine blue. Red panda eMSCs cultured in normal growth medium served as control.

For hepatogenic differentiation, eMSCs were cultured in hepatogenic differentiation medium (Cyagen) as per the manufacturer’s protocol. The medium was changed three times per week. After 16 days, hepatogenic differentiation was evaluated by cytokeratin 18 (CK18) (ab181597, Abcam) immunofluorescence staining, detection of *CK 18*, *ALB* and *DKK1* mRNA expression, ALB protein expression, Periodic Acid-Schiff (PAS) staining and Indocyanine Green (ICG) uptake. Red panda eMSCs cultured in normal growth medium served as control.

### PAS staining

Red panda eMSCs after hepatogenic differentiation were stained by PAS staining kit (Solarbio). Following the manufacturer’s instructions, hepatogenic differentiated cells were washed twice with PBS, then fixed with 4% paraformaldehyde (Sigma) for 15 min. Cells were incubated in 1% periodic acid for 20 min and then washed four times with distilled water. After incubating with Schiff regent for 20 min, the cells were counterstained with Mayer haematoxylin solution for 2 min. Then, the stained cells were rinsed in distilled water and photographed under an inverted microscope (IX73, Olympus).

### ICG uptake

Red panda eMSCs after hepatogenic differentiation were determined through detecting the cellular uptake of ICG. Briefly, hepatogenic differentiated cells were washed twice with PBS, then incubated with LG-DMEM supplemented with 1 mg/ml of ICG (Sigma), and incubated at 37°C with 5% CO_2_ for 1 h. Subsequently, the cells were washed three times with PBS, and then photographed under an inverted microscope (IX73, Olympus).

## Statistical analysis

The results of growth curves were analysed by using GraphPad Prism 5 software (GraphPad Software, San Diego, CA, USA). Data were expressed as the mean ± SEM. All results were generated from at least three independent experiments.

**Figure 1:**
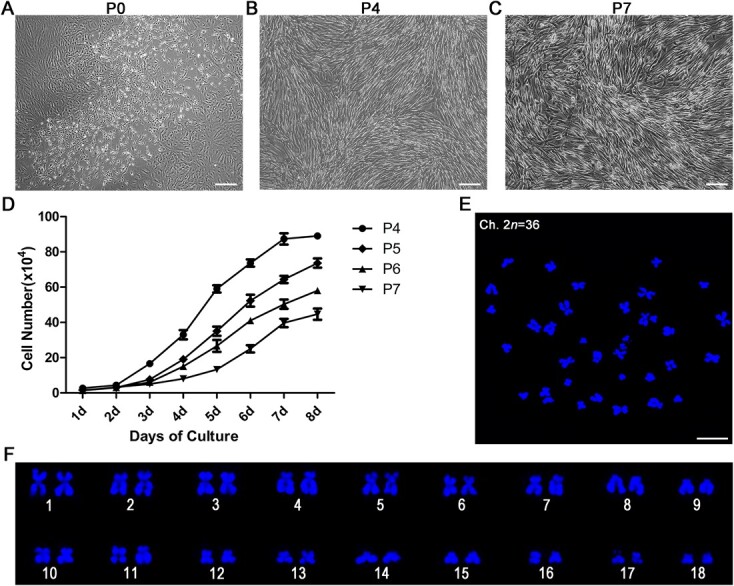
Morphological characteristics, growth curves and chromosome spreads of red panda eMSCs. (**A**) Representative image of primary red panda eMSCs after 9 days culture. The primary cells were fibroblast like and epithelial like, presenting triangular, fusiform, ovoid or polygonal shapes. Scale bar, 200 μm. (**B**) Representative image of red panda eMSCs at passage 4. Fibroblast-like shape and presented polygonal or long spindle shape. Scale bar, 100 μm. (**C**) Representative image of red panda eMSCs at passage 7. Cells still maintain fibroblast-like and long spindle shapes. Scale bar, 100 μm. (**D**) The growth curves of the cells from passages 4–7. Quantified data show the mean ± SEM. (**E**) Chromosome spreads of red panda eMSCs at passage 8. Scale bar, 10 μm. (**F**) Analysis of the chromosome spreads of (E). The correct number of chromosomes in red panda is 2*n* = 36.

## Results

### Isolation and culture of red panda eMSCs

After 9 days culture, the primary cells were fibroblast like and epithelial like, presenting triangular, fusiform, ovoid or polygonal shapes, and the overall percentage of eMSCs was ~50% (Fig. 1A). Red panda eMSCs at passage 4 were fibroblast like and presented polygonal or long spindle shapes (Fig. 1B). After seven generations, eMSCs also have the ability to maintain their morphological characteristics (Fig. 1C). The growth curves of red panda eMSCs from passage 4 to 7 were established. Results showed that eMSCs entered the exponential phase after 4 days culture and then reached the stationary phase after 7 days culture (Fig. 1D). After culturing eight passages, the chromosome number of red panda eMSCs are still normal (2*n* = 36) (Fig. 1E and F).

### Surface antigens and transcription factors in red panda eMSCs

**Figure 2:**
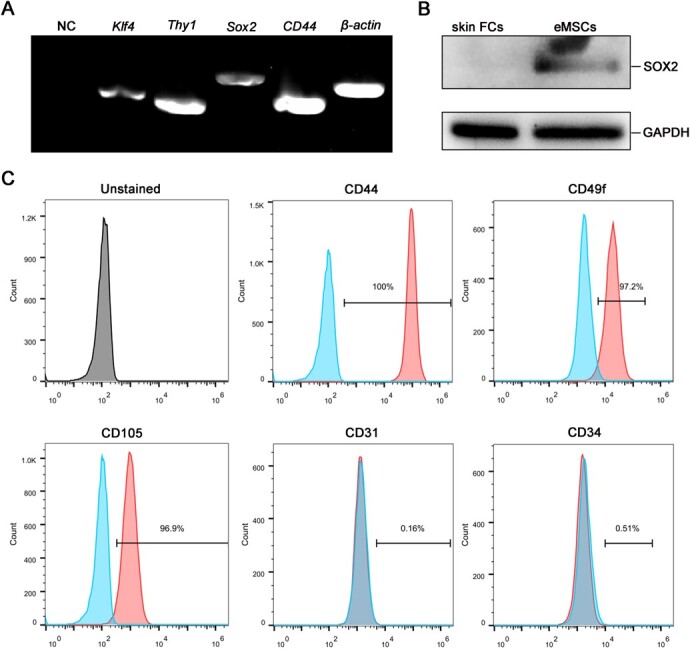
Characterization of red panda eMSCs. (**A**) RT-PCR analysis of pluripotency genes in eMSCs. *β-actin* was used as a control for RNA sample quality. (**B**) The expression of SOX2 was detected in red panda eMSCs and skin FCs by western blot. GAPDH was used as the loading control. (**C**) Red panda eMSCs were stained with phycoerythrin (PE)-conjugated CD44, CD49f, CD105, CD34 or PE-Cyanine7 conjugated CD31 antibodies. Blue areas, signal from isotype controls; red areas, signal from the specific cell surface marker; grey areas, unstained black control.

The expressions of pluripotency and MSC marker genes, including *Thy1*, *Klf4*, *Sox2* and *CD44*, were confirmed by RT-PCR. Genes *Thy1*, *Klf4*, *Sox2* and *CD44* were all highly expressed in red panda eMSCs (Fig. 2A). Additionally, SOX2 protein was also detected in eMSCs, but not in skin FCs (Fig. 2B). According to the flow cytometry analysis, the red panda eMSCs were positive for MSCs phenotype CD44, CD49f and CD105 and negative for endothelial cell marker CD31 and haematopoietic cell marker CD34 (Fig. 2C).

### Differential gene expression analysis between red panda eMSCs and skin FCs

**Figure 3:**
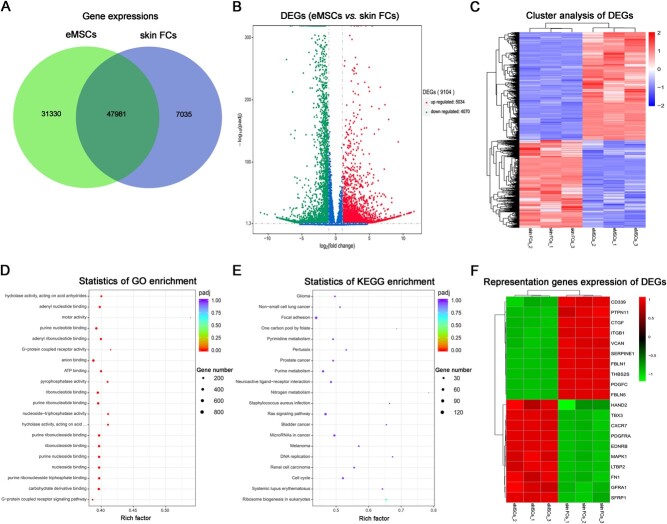
Differential gene expression analysis between red panda eMSCs and skin FCs. (**A**) The Venn diagram of the number of predicted genes in red panda eMSCs and skin FCs. (**B**) Volcano map of DEGs between red panda eMSCs and skin FCs. The x-axis is the log2 scale of the fold change of gene expression in eMSCs and skin FCs (log_2_(fold change)). Negative values indicate downregulation; positive values indicate upregulation. The y-axis is the minus log10 scale of the adjusted *P*-values (−log_10_(padj)), which indicate the significant level of expression difference. The red dots represent significantly upregulated genes with at least 2-fold change, while the green dots represent significantly downregulated genes with at least 2-fold change. (**C**) Heatmap of the differentially expressed genes between red panda eMSCs and skin FCs. Red stripes represent high expression genes, while blue stripes represent low expression genes. (**D**) Top 20 significantly enriched GO terms. (**E**) Top 20 enriched KEGG pathways. (**F**) Heatmap of the representative functional genes expression of DEGs between red panda eMSCs and skin FCs. Red stripes represent high expression, while green stripes represent low expression.

A total of 79 311 and 55 016 predicted expressed genes were identified in eMSCs and skin FCs, respectively. Of these, 47 981 genes were common expressed in eMSCs and skin FCs (Fig. 3A). Compared with skin FCs, 5034 genes upregulated in eMSCs and 4070 genes downregulated (Fig. 3B), and DEGs could significantly separate the samples into the eMSCs group and skin FCs (Fig. 3C). DEGs were analysed by GO annotation, and the significantly enriched GO terms mainly contained G-protein coupled receptor signalling pathway, carbohydrate derivative binding, nucleoside binding, purine nucleoside binding, ribonucleoside binding, motor activity, etc. (Fig. 3D). The signal pathways of DEGs were analysed by KEGG pathway analysis. As shown in Fig. 3E, the top 20 mainly enriched KEGG pathways were ribosome biogenesis, cell cycle, DNA replication, Ras signalling pathway, purine metabolism, etc. In addition, some representative genes about promoting MSCs differentiation and proliferation were upregulated and promoting fibroblasts proliferation were downregulated in eMSCs group when compared skin FCs. Also, all these genes significantly separated the samples into the eMSCs group and skin FCs (Fig. 3F; Table 3).

**Table 3:** Representation of 10 upregulated genes and 10 downregulated genes in red panda eMSCs compared with skin FCs

Gene name	Expression	log_2_FC	*q* value	Gene description
*PDGFRA*	Upregulated	3.013	0.000	Directs eMSCs differentiation towards chondrocyte progenitor fate (Bartoletti *et al.,* 2020)
*TBX3*	Upregulated	1.298	0.000	Plays an important role in osteogenic differentiation and proliferation of human mesenchymal stem cells derived from adipose tissue (Lee *et al.,* 2007)
*CXCR7*	Upregulated	3.584	0.000	Critical regular for MSC-mediated vasculogenesis (Wei *et al.,* 2020)
*EDNRB*	Upregulated	5.457	0.000	Direct bone marrow-derived hMSCs for osteo- and chondro-lineage differentiation (Lee *et al.,* 2019)
*MAPK1*	Upregulated	1.023	0.000	Promote osteogenesis and angiogenesis (Bian *et al.,* 2019)
*GDNFRA*	Upregulated	2.600	0.000	Improve the efficiency of MSC in the recovery of kidney (Esmaeilizadeh *et al.,* 2020)
*SFRP1*	Upregulated	6.128	0.000	Regulate the proliferation and differentiation of bone mesenchymal stem cells (Tang *et al.,* 2019)
*FN1*	Upregulated	1.602	0.000	Regulate myofibroblast differentiation of endometrial MSCs (Zhang *et al.,* 2019)
*LTBP2*	Upregulated	6.092	0.000	Expressed primarily in cell types of mesenchymal origin, particularly osteoblasts and chondrocytes (Davis *et al.,* 2014)
*HAND2*	Upregulated	4.547	0.000	Establishing distinct mesenchymal compartments (Osterwalder *et al.,* 2014)
*SERPINE1*	Downregulated	−6.749	0.000	Contribute to epithelial dedifferentiation, G2/M proliferative arrest, fibrogenesis (Gifford *et al.,* 2021)
*PDGFC*	Downregulated	−1.678	0.000	Induce progressive fibrosis (Wright *et al.,* 2014)
*FBLN1*	Downregulated	−4.985	0.000	Associate with lung fibrosis in both humans and mice and stabilizes collagen formation (Liu *et al.,* 2019)
*THBS2S*	Downregulated	−1.455	0.000	A key regulator of fibrosis (Reinecke *et al.,* 2013)
*CTGF*	Downregulated	−3.648	0.000	Associate with atrial fibrosis (Chen *et al.,* 2019)
*JAGGED*	Downregulated	−2.487	0.000	Play a key role in tissue fibrosis (Zhao *et al.,* 2018)
*PTPN11*	Downregulated	−2.242	0.000	Associate with fibrosis promotion during the early stages of HCC development (Kang *et al.,* 2017)
*ITGB1*	Downregulated	−1.811	0.000	Associate with proliferation and migration of fibroblasts (Baszanowska *et al.,* 2021)
*VCAN*	Downregulated	−1.766	0.000	Affect phenotype of cultured human dermal fibroblasts (Merrilees *et al.,* 2016)
*FBLN5*	Downregulated	−4.630	0.000	Affect adhesion and proliferation of human fibroblast-like cells (Furie *et al.,* 2016)

### Differentiation of red panda eMSCs

**Figure 4:**
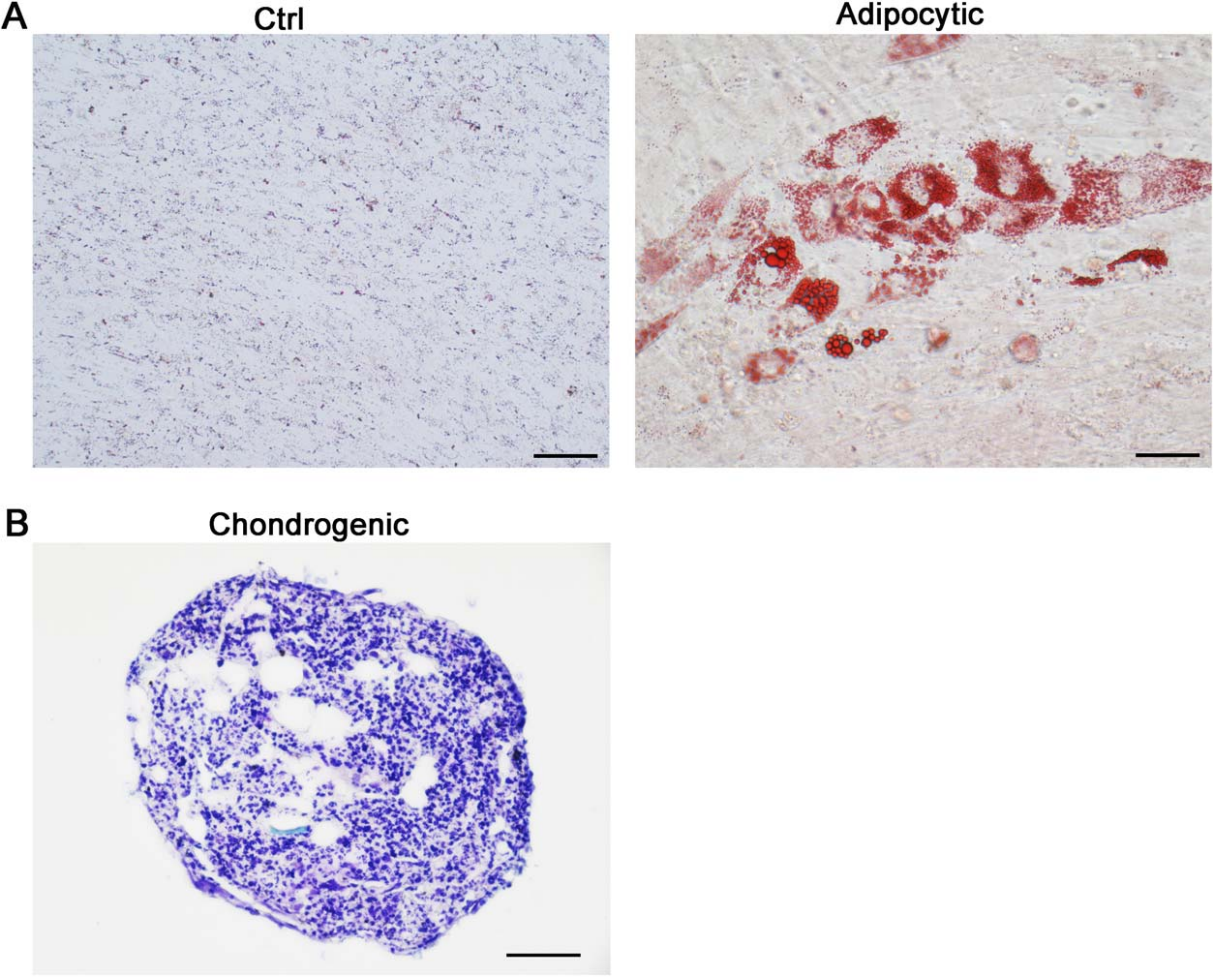
Adipocytic and chondrogenic differentiation of red panda eMSCs. (**A**) Red panda eMSCs were cultured in the adipocytic induction medium for 8 days, and then evaluated by Oil-red O staining to reveal lipid vacuoles. Untreated cells were used as control. Scale bar, 50 μm. (**B**) Red panda eMSCs were cultured in the chondrogenic differentiation medium for 21 days, and then evaluated by toluidine blue staining to reveal the sulfated proteoglycans of the cartilage matrices. Scale bar, 50 μm.

For adipocytic differentiation, cells were cultured in adipocytic induction medium. In the control group, no positive staining signal of Oil-red O was detected (Fig. 4A). In the induced group, many lipid droplet accumulations were detected by the staining of Oil-red O (Fig. 4B).

For chondrogenic differentiation, cells were cultured in chondrogenesis differentiation medium to form chondrogenic pellets. The toluidine blue staining was used to evaluate the sulfated proteoglycans of the cartilage matrices. The results showed positive staining signals (Fig. 4C).

**Figure 5:**
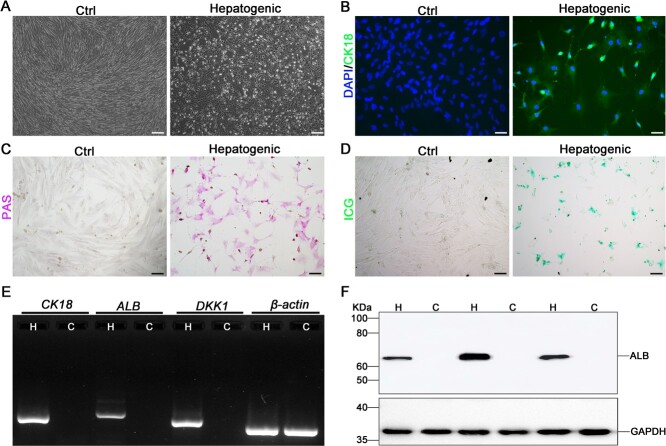
Hepatogenic differentiation of red panda eMSCs. (**A**) Bright field images of red panda eMSCs cultured in hepatogenic differentiation medium for 16 days, and eMSCs were changed to flat polygonal morphology. Untreated cells were used as control. Scale bar, 200 μm. (**B**) Red panda eMSCs after hepatogenic differentiation were stained with anti-CK18 (hepatogenic marker, green), and the nucleus were stained with DAPI (blue). Untreated cells were used as control. Scale bar, 50 μm. (**C**) Red panda eMSCs after hepatogenic differentiation were stained with PAS staining (purple signal). Untreated cells were used as control. Scale bar, 100 μm. (**D**) Red panda eMSCs after hepatogenic differentiation were detected by ICG uptake (green signal). Untreated cells were used as control. Scale bar, 100 μm. (**E**) The expressions of liver-specific genes in hepatogenic-induced (H) and control (C) red panda eMSCs were detected by using RT-PCR. *β-actin* was used as a reference gene. (**F**) The expressions of ALB in hepatogenic-induced (H) and control (C) red panda eMSCs were detected by using western blot. GAPDH was used as the loading control.

To determine whether red panda eMSCs have the ability to endoderm differentiation, cells were cultured in hepatogenic differentiation medium. As shown in Fig. 5A, the cells after differentiating became flat, presenting polygonal morphology when compared with the control group. Immunofluorescence staining revealed strong positive signals of the hepatogenic marker CK18 in the hepatogenic-induced group, but no positive staining was detected in the control group (Fig. 5B). The results of PAS staining showed that extensive cytoplasmic positive staining signalling (red to purple) were only observed in the hepatogenic-induced group (Fig. 5C). The hepatogenic differentiated group successfully showed an indocyanine green uptake staining and the control group could not upake ICG (Fig. 5D). In addition, the results of RT-PCR further showed that the hepatic and liver progenitor marker genes *CK18*, *ALB* and *DKK1* were highly expressed in the hepatogenic-induced group when compared with the control group (Fig. 5E). And the result of western blot also showed that the ALB was highly expressed in the hepatogenic-induced group when compared with the control group (Fig. 5F).

## Discussion

In the present study, we successfully isolated eMSCs in red panda endometrium. The primary cells were fibroblast like and epithelial like, presenting triangular, fusiform, ovoid or polygonal shapes, because primary cells were a mixed-cell group, containing epithelial cells, basal layer cells and eMSCs. After passage cultivations, fibroblast-like eMSCs gradually became the major cells, suggesting that the currently used culture method would greatly benefit eMSCs growth and reproduction. During proliferation in eight successive days, eMSCs at passage 4 experienced a short lag phase from Days 1 to 4 and subsequently a logarithmic rise from Day 4, then reached the stationary phase at Day 7 (Fig. 1). However, eMSCs at passages 5–7 did not reach the plateau phase at Day 8. It may be that the cells did not reach the maximum growth density. The eMSCs at passage 7 still had a strong proliferative potential, suggesting eMSCs had strong proliferation stability. These results were similar to the growth curves of mesenchymal stem cells isolated from red panda bone marrow (An *et al.,* 2020). Red panda eMSCs at passage 8 showed normal diploid karyotype (2*n* = 36), suggesting that this type of cell had a stable and normal growth and reproduction, as well as further confirmed that the currently used culture method was appropriate.

To characterize the red panda eMSCs isolated in present study, we examined some MSCs biomarkers, such as CD44, CD49f, CD90 and CD105. The red panda eMSCs highly expressed CD44, CD49f, CD105, but not expressed CD31 and CD34 when compared with the isotype control. The results of RT-PCR also confirmed CD90 (Thy1) was highly expressed in red panda eMSCs. These results suggested that the red panda eMSCs were neither endothelial cell nor haematopoietic cell and was in accord with the mesenchymal cells standards of the International Society for Cell Therapy (Dominici *et al.,* 2006). Pluripotency genes *Sox2* and *Klf4* also expressed in red panda eMSCs. However, other markers THY1 and OCT4 were not detected with western blot analysis, this may be because there are no specific antibodies available for red panda or the expression of these markers in red panda eMSCs is too low to be detected. In the future studies, more specific antibodies to recognize red panda should be selected and further supplement the current study.

In order to analyse the red panda eMSCs characters in gene expression profile, RNA-seq analysis between eMSCs and skin FCs was performed. To date, the complete gene expression profile of red panda has not yet been established, thus the present study adopted a no reference transcriptome analysis, which might lead to an incomplete functional genes annotation. We used Trinity software (Grabherr *et al.,* 2011) to identify 86 346 unigenes (genes), and the number of genes was different from referenced mammalian animals. We identified 9104 genes that were differentially expressed. Moreover, the top 20 enrichment pathways of DEGs in GO and the KEGG mainly associated with G-protein coupled receptor signalling pathway, carbohydrate derivative binding, nucleoside binding, ribosome biogenesis, cell cycle, DNA replication, Ras signalling pathway, purine metabolism and cell cycle. These results suggested that eMSCs had a high frequency of cellular activity and proliferative capacity. Among the DEGs, some genes about promoting MSCs differentiation and proliferation were upregulated and promoting fibroblasts proliferation were downregulated in eMSCs group (Table 3), which further confirmed the eMSCs pluripotency.

MSCs have the ability to multiple differentiation, and earlier studies had reported that MSCs could differentiate into adipocytes, osteoblasts, chondrocytes, neural cells and smooth muscle cells (Liechty *et al.,* 2000; Wu *et al.,* 2007). In the present study, we confirmed the red panda eMSCs could be differentiated into adipocytes and chondrocytes, from which were mesoderms. Additionally, the red panda eMSCs also had the ability to differentiate into hepatocytes, from which were endoderms. This result revealed that the red panda eMSCs have the capacity for differentiation potential across embryonic lineage boundaries. Interestingly, previous research had shown that MSCs from bone marrow could differentiate into hepatocytes *in vitro* (Schwartz *et al.,* 2002). Moreover, some studies revealed that eMSCs are likely to be derived from bone marrow (Cervello *et al.,* 2012; Cervello *et al.,* 2015), but the real origin of red panda eMSCs needs further studies.

The red panda eMSCs with high proliferation *in vitro*, stable karyotype and multipotential differentiation have more potential applications in wildlife protection and future research and/or clinical practice. Firstly, somatic cell nuclear transfer (SCNT) or interspecies SCNT (iSCNT) may be a potential tool for aiding the conservation of endangered animal species, although accompanied with low efficiency and mitochondrial heterogeneity, which could compromise the energy-making process of embryo, leading to its death (Loi *et al.,* 2011). To date, iSCNT has been successfully performed in many endangered wild animals, such as African wildcat (*Felis lybica*) (Gomez *et al.,* 2004), Bactrian camel (*Camelus bactrianus*) (Wani *et al.,* 2017) and even the extinct species Pyrenean ibex (*Capra pyrenaica pyrenaica*) (Folch *et al.,* 2009). A previous study tried to construct interspecies cloned embryos by using red panda fibroblasts and rabbit enucleated oocytes (Tao *et al.,* 2009). It is well known that fibroblasts were common donor cells used in SCNT, but MSCs were also suitable. In 2018, horse MSCs from bone marrow were used as nuclear donor cells and produced healthier cloned horses compared with fibroblasts (Olivera *et al.,* 2018). Red panda eMSCs derived from endometrium, which directly interacted with early embryos, may be more suitable and further increase the SCNT embryo development. Secondly, to date, embryonic diapause occurs in over 130 species of mammals (Deng *et al.,* 2018) including red panda (Macdonald *et al.,* 2010; Miles *et al.,* 1979), but the potential mechanism is still unclear. Embryo implantation is a key step in the establishment of pregnancy, and the eMSCs is a great cell model for studying embryonic diapause. On one hand, endometrium of mammal secretes cytokines and growth factors that influence the development of early embryo (Cha *et al.,* 2012). It is likely that some of growth factors control the arrested growth that occurs in diapause (Renfree & Fenelon, 2017). It is also clear that MSCs secrete a variety of cytokines and growth factors, such as insulin-like growth factor-1, vascular endothelial growth factor, epidermal growth factor, fibroblast growth factor, interleukin-6, leukaemia inhibitory factor and transforming growth factor-β (Feng *et al.,* 2009). Therefore, it will be a new angle to study embryonic diapause from the eMSCs secretion. On the other hand, during embryo implantation, there is intercellular communication between the embryo and maternal eMSCs and peripheral blood MSCs (pbMSCs), which could chemotax to embryonic trophectoderm secretome (Calle *et al.,* 2021a, Calle *et al.,* 2021b). In bovine embryo implantation, the migratory capacity of eMSCs was increased towards an inflammatory niche and then reduced by the expression of implantation cytokine by the embryo, which are necessary for immunorepression to prevent embryo rejection by the maternal organism (Calle *et al.,* 2019). Therefore, deeply to study the communication between eMSCs and embryo will be beneficial for clarifying the regulation mechanism of embryo implantation. Finally, early embryo mainly develops in the maternal oviduct and uterus, which contains a large number of factors that can promote early embryo development (Kolle *et al.,* 2020). Recent studies found that coculture of mouse embryos and mesenchymal stem/stromal cells derived from menstrual blood enriched the embryonic microenvironment and promoted embryo development (Goncalves *et al.,* 2020). Coculture with extracellular vesicles from endometrial-derived MSCs could increase the quality of aged mouse embryos and presumably by modulating the expression of antioxidant enzymes and promoting pluripotent activity (Marinaro *et al.,* 2019). Therefore, paracrine regulation of eMSCs may be a feasible way to promote *in vitro* embryo development, which will benefit endangered wild animals like the red panda.

## Conclusion

In this study, we, for the first time, isolated and characterized the red panda eMSCs in endometrium. Red panda eMSCs were fibroblast like and highly expressed the pluripotency genes including *Thy1*, *Klf4* and *Sox2*. Additionally, red panda eMSCs were positive for MSC markers CD44, CD49f and CD105 and negative for endothelial cell marker CD31 and haematopoietic cell marker CD34. The red panda eMSCs also had the pluripotent differentiation capacities of adipocytes, chondrocytes and hepatocytes. Using RNA-seq, significant DEGs were identified, which further demonstrated the eMSC gene expression characters. The new multipotential stem cell could not only benefit the germ plasm resources conservation of red panda, but also basic or pre-clinical studies in the future.

## Funding

This work was supported by the Sichuan Science and Technology Program (2020JDJQ0074); the Chengdu Research Base of Giant Panda Breeding (2020CPB-B07); and the Chengdu Giant Panda Breeding Research Foundation (CPF2017–16).

## Supplementary material

Supplementary material is available at *Conservation Physiology* online.

## Conflict of interest

The authors declare no conflict of interest.

## Authorship contribution statement

Dong-Hui Wang: conceptualization, methodology, writing (original draft), review and editing. Xue-Mei Wu: visualization, investigation and writing (original draft). Jia-Song Chen, Yuan Li and Fei-Ping Li: investigation. Zhi-Gang Cai, Jun-Hui An and Ming-Yue Zhang: formal analysis. Rong Hou: project administration. Yu-Liang Liu: conceptualization, methodology, review and editing.

## Data availability

The data underlying this article are available in the article and in its online supplementary material.

## Supplementary Material

Table_S1_coac004
