# Supplementary material for: Isolation and characterization mesenchymal stem cells from red panda (Ailurus fulgens styani) endometrium
Source: Conserv Physiol. 2022 Feb 21;10(1):coac004. doi: 10.1093/conphys/coac004 (PMC8862722; doi:10.1093/conphys/coac004)
Supplement: Table_S1_coac004 [file table_s1_coac004.docx]

**Table S1**

| **Individuals** | **Age** | **Estrous cycle stage** |
| --- | --- | --- |
| Red panda 1 | 2 months | not sexually matured |
| Red panda 2 | 2 months | not sexually matured |
| Red panda 3 | 5 months | not sexually matured |
| Red panda 4 | 6 months | not sexually matured |
| Red panda 5 | 3 years | diestrus |
| Red panda 6 | 5 years | diestrus |

Detail information of six red pandas
